# Transcendental Meditation Enriches Nurses’ Authentic Presence Through Caring for Self and Others

**DOI:** 10.1177/08980101241262922

**Published:** 2024-07-26

**Authors:** Catherine Aquino-Russell, Jennifer I Bonamer, Susan Hartranft, Mary Kutash, Ayesha Johnson

**Affiliations:** 3427 University of New Brunswick; 7226 Sarasota Memorial Hospital; 25301 Moffitt Cancer Center; 7829 Tampa General Hospital; 7831 University of South Florida

**Keywords:** clinical nurses, well-being, authentic presence, present moment awareness, transcendental meditation, self-care

## Abstract

**Purpose of Study:** Given the enormity of the most recent challenges to clinician well-being, intensified by the pandemic, we decided to explore the influence of Transcendental Meditation^®^ (TM)^®^ on the well-being of clinical nurses. The purpose of our study was to use qualitative analysis to enhance our understanding of the experiences of clinical nurses who practiced TM, as viewed through the lens of our conceptual model and Watson's holistic unitary caring science theory. **Design and Method:** This qualitative study involved a thematic analysis of clinical nurses’ written descriptions following the completion of the TM program during the COVID-19 pandemic. **Findings:** The nurse participants shared their experiences with the practice of TM as creating present moment focus, leading to enhanced self-care, and development of authentic presence with others. The overall theme uncovered in the analysis is that authentic presence is veritas (truth) in knowing, being, doing, and becoming. **Conclusions:** The findings were congruent with Watson's unitary caring science theory and provided illumination of the holistic value of TM as a self-care strategy for supporting nurses’ well-being with the goal of retaining nurses in practice. When nurses care for themselves, they are more likely and able to care for others, thus helping them to enjoy their nursing careers.

## Background

The National Academy of Medicine (NAM) has highlighted the health and well-being of healthcare workers, including clinical nurses, because of the greater risk for burnout, depression, compassion fatigue, and poor work–life balance (NAM, 2020). With the COVID-19 pandemic, the stress on nurses was magnified (American Nurses Foundation, 2022; Guttormson et al., 2022). A healthy nursing workforce has beneficial implications for patients, nurses, and organizations (Melnyk et al., 2018). However, even in the best of times, nurses may not prioritize caring for their own multidimensional health and well-being., that is, physical, mental, emotional, social, and spiritual. Consistent with the core values of holistic nursing practice, self-care is an essential component of nurses’ journey toward well-being (ANA & AHNA, 2019). An evidence-based strategy to support health and well-being across populations, including nurses, is Transcendental Meditation (TM; Azizoddin et al., 2021; Bonamer & Aquino-Russell, 2019; Bonamer et al., 2024; Calarco & Stratton, 2023, Nestor et al., 2023; Perkins & Aquino-Russell, 2017). This article describes qualitative findings from a multimethod study of clinical nurses practicing TM during the COVID-19 pandemic.

## Literature Review

For over 60 years, rigorous published research on the benefits of TM for mental, physical, and social well-being has been conducted among clinicians, students, veterans, and others of all ages, nationalities, and religions (Aquino-Russell et al., 2023; Bellehsen et al., 2022; Chalmers et al., 1989; Orme-Johnston & Barnes, 2014; Schneider et al., 1995, 2022; Wallace et al., 1990). TM is a simple, natural technique practiced 20 minutes twice each day while sitting comfortably with the eyes closed. It is easily learned, and is not a religion, philosophy, or lifestyle. It does not involve concentration, control of the mind, contemplation, or monitoring of thoughts or breathing. The practice allows the active thinking mind to settle down to a state of inner calm (Transcendental Meditation for Nurses, 2024).

Among meditation practices, the TM technique is unique because it facilitates automatic self-transcending as compared to others, which may incorporate focused attention or open monitoring (Travis & Shear, 2010). Automatic self-transcending requires minimal cognitive control (Travis & Shear, 2010), and does not include mindfulness, concentration, focus on breathing, or trying to “clear your mind” (Maharishi Foundation International, 2024).

The TM for Nurses Program was implemented specifically for the promotion of TM among nurses and provided strategic and logistical support for the conduct of this study (Transcendental Meditation for Nurses, 2024). In partnership with TM for Nurses, findings from an earlier study of the use of TM among clinical nurses demonstrated the potential for improvement in well-being, that is, resilience and compassion fatigue (Bonamer & Aquino-Russell, 2019). More recently, improvements were noted in burnout (emotional exhaustion), anxiety, and mental well-being (Bonamer et al., 2024; Calarco & Stratton, 2023), depression (Calarco & Stratton, 2023), and PTSD (Bonamer et al., 2024).

The NAM conceptual model of factors affecting clinician well-being and resilience describes coping and resilience skills which lead to improvements in personal factors such as well-being (NAM, 2020). This NAM model became part of our study's conceptual model as TM has the potential to fortify some of these skills and abilities.

## Conceptual Model

The conceptual model for this study was an integration of both the NAM's factors affecting clinician well-being and resilience model (NAM, 2020) and the veterans’ administration circle of health (VA, 2020). Our conceptual model for this study involves three embedded concentric circles. The model is nurse-centric with the core representing present moment awareness. We thought that nurses’ awareness would influence their well-being, which is the middle circle. We considered that well-being influences the capacity of the nurse to experience their authentic presence with self/others, as seen in the third concentric or outer circle. Our study's inclusion of authentic presence was informed by Watson's theory of unitary caring science and the 10 caritas processes^®^ (ANA & AHNA, 2019; Watson, 2018). For example,

Presence cannot be known from the outside in but only from the inside out, from the subjective life experience of the people in any given moment. Authentic presence in a given moment between persons that captures the human-to-human, spirit-to-spirit connection, which is felt experientially but may not be detected by an outside objective observer. (Watson, 2018, p. 90)

In relation to our conceptual model, we believe that nurses must have present-moment awareness, which enables well-being and leads to authentic presence with others as an evolving process from the inside out.

## Purpose

Given the enormity of the most recent challenges to clinician well-being, intensified by the pandemic, we decided to explore the influence of TM on the multidimensional well-being of clinical nurses using both qualitative and quantitative approaches. For quantitative findings, please refer to Bonamer et al. (2024). The purpose of the qualitative component of our study was to use thematic analysis (TA) to enhance understanding of the well-being experiences of clinical nurses who practiced TM, in light of our conceptual model which reflected elements of Watson's (2018) unitary caring science theory.

## Method

The overall multimethod study involved qualitative TA and a randomized controlled trial to describe/evaluate the well-being of clinical nurses following the instruction and practice of TM during the pandemic. As part of the study, participants were provided TM instruction. Surveys were conducted at baseline, 1-month and 3-months; however, the qualitative component was only included in the 3-month survey. Following submission of the baseline survey, participants were randomized to either immediate instruction (intervention group) or delayed instruction which happened after the final 3-month survey (control group). The qualitative component of our study involved TA to describe the experiences of clinical nurses following the instruction and practice of TM during the COVID-19 pandemic.

### Participants/Setting (Selection and Description)

Participants included clinical nurses (RN, LPN, and APRN) from three Magnet designated hospitals in southwest Florida during the pandemic (2020/2021). Each hospital had a clinical nurse researcher who served as the site principal investigator. Inclusion criteria involved the ability the read and write in English and being stable on any medications for at least 2 months. Exclusion criteria included individuals with prior TM training because training includes lifetime follow-up, and therefore they would not be eligible for repeating initial instruction. No limitations or assessments were provided for exposure to other meditation approaches.

### Ethical Considerations

Participation was voluntary, with the choice to respond or not respond to the questionnaire. The site principal investigators (JB, SH, MK) used a participant information sheet to provide informed consent both verbally and in writing. To protect confidentiality, only the lead study principal investigator (JB) had the record linking personal identifiers to participants’ responses. Participants’ written descriptions were coded by study numbers rather than by names for protection of participant anonymity. The study team considered the participants to be more vulnerable due to the challenges of caring for patients during the COVID-19 pandemic, so we were intentional in offering respect, caring support, and freedom to withdraw at any time from the study with no repercussions.

Institutional Review Board (IRB) approval was obtained through the principal investigator's (JB) hospital (expedited review), which served as the IRB of record for the other associated hospitals in Florida. Review and approval were also obtained through the qualitative researcher's (CA-R) university in Canada.

### TM Instruction Intervention

Certified TM instructors provided the standard TM training course to participants in the immediate instruction group with the expectation of continued practice twice daily for 20 minutes. Initial training included four consecutive days with follow-up support offered to participants at 2 weeks, 1-, 2-, and 3-months. Of note, the American Holistic Nurses Association has approved the TM for Nurses program for continuing education credit upon successful completion (TM-Nurses.org).

### Instrumentation

The demographic questions collected participants’ personal and professional information, including age, years in nursing practice, hospital, and type of practice area. The qualitative questionnaire was embedded in the 3-month survey completed by those nurses who received immediate TM instruction. It was designed from the conceptual model and guidance from Watson's (2018) unitary caring science theory utilizing open comment boxes. Participants responded to focused questions related to their awareness of self, domains of well-being, and authentic presence with self and others, using their TM practice as a frame of reference.

### Procedures for Data Collection

Data were collected by email invitations with an embedded link to a survey in REDCap, which stands for research electronic data capture (Harris et al., 2009, 2019). Participants completed the demographic questions at baseline and the qualitative questionnaire at Month 3.

### Qualitative Approach

Qualitative TA was used to analyze qualitative questionnaire responses. TA is “the analysis of narrative data [or in this case, written descriptions] to identify prominent themes and patterns among the themes” (Loiselle et al., 2007, p. 395). This method is suitable for studying phenomena when little knowledge is available, as it helps to elucidate and deepen the understanding of concepts and meaning (themes) associated with the experience (Braun & Clarke, 2012; Loiselle et al., 2011). “Through focusing on meaning across [written descriptions], TA allows the researcher to see and make sense of collective or shared meanings and experiences” (Braun & Clarke, 2012, p. 57). Steps of TA, as described by Erlingsson and Brysiewicz (2017), were utilized. We began the TA with multiple readings of the written descriptions to gain a thorough understanding of each participant’s perspectives and main ideas. Next, we color-coded participants’ ideas/words, and then consolidated the color-coded content into themed categories. Across question responses, we developed overarching themes to capture the essence or meaning of participants’ experiences as guided by Watson's (2018) unitary caring science theory.

## Results

Of the 104 clinical nurses enrolled in this study, 53 nurses were randomized to receive immediate instruction within the year November 2020–2021. They were mostly RNs (92%), female (98%), and Caucasian (72%), with an average age of 42 years and a mean of 12.5 years in practice as nurses. Of the 53 participants, 46 (87%) of them completed the initial 4-day training plus at least two of the additional follow-up sessions. Nurses in the study were from three different clinical locations and provided individual and assigned group TM instruction at different times according to their enrollment date and availability. The monthly average of adherence to at least daily practice most days of the week was self-reported by over 90% of participants. Due to the voluntary nature of our survey, qualitative responses were provided by 44 of these participants (96%). Next, we will present our qualitative findings.

### Qualitative Analysis

Through our analysis, we uncovered the following themes of meaning for our participants’ transformational experiences from practicing TM during the COVID-19 pandemic. Below we will present the themes and subthemes as well as participants’ direct quotations (in italics) to exemplify our findings.

Across participants’ descriptions of present moment awareness, domains of well-being, and authentic presence with self and others surfaced the following overall essence:

**Authentic presence is veritas (truth) in knowing, being, doing, and becoming.**
  - **Knowing is awareness of truth in the moment for self/others.** This theme demonstrates the primacy of participants in noticing self and others with intention.
  - **Being is caring for self as truth.** This theme describes how participants built on their awareness to *carefully* create space for their own well-being.
  - **Doing is living truth through cocreating a healing environment with others.** This theme reflects the outward intention of engaging with others using self as a healing environment.
  - **Becoming is connection as universal truth through transcending self.** This theme reveals participants’ descriptions of ascending toward a holistic perspective of oneness.

#### Themes, Subthemes, and Participants’ Direct Quotations

##### Knowing is awareness of truth in the moment for self/others

###### Developing Greater Self-Awareness

- More aware of my reaction to almost any situation, good or bad. More aware of my weaknesses and strengths. Way more aware of what is specifically causing me challenges.
- Being aware of how different emotions show up in my body.
- Figuring out who I am in comparison to the voice in my mind.
- I was always in an emotion but not centered. My words and thoughts were tethered to the emotion, and I was never aware of the emotion.
- I observe body language, others and my own. TM has helped me become more aware of my actions and reactions when I am interacting with others.

###### Being Present or Mindful in the Moment With Self/Others Without Distraction

- I appreciate the attention (TM has) brought to living in the moment.
- … without having your mind on other things … Being mindful of the experience, you are in at that moment.
- I take more time with others, to listen with my full attention, tune into the feelings that arise in my body during different situations and with different people.

##### Being is caring for self as truth

###### Caring for Integrity of Self

- I am able to say “no” more often or “sorry, I can't do that” and not be guilted into changing my mind. I am able to listen to the boundaries of others who are saying “no” or “I can’t do that.”
- I have felt more confident in myself and my needs as a person and not just an employee. It has helped me advocate for myself and what I deserve.
- I have found that being more authentic around others has been healing to me as I am being accepted for who I am.

###### Prioritizing Self-Care for Well-Being

- I realize that I need to care for myself in order to better care for my family and patients.
- Taking time for myself enables me to see solutions and options more clearly.

###### Strengthening Physical Well-Being

- TM has given me an outlet to recharge my batteries … more energy.
- TM has helped to improve my sleep and I feel like I can get to sleep easier as well as feel more rested when I wake up.
- I am aware that I need to take more time for myself. Not only to meditate, but I’ve noticed that I have prioritized myself. For example, I am taking time to exercise.
- TM has brought me a greater awareness of what I need for myself physically and mentally to be able to relax and recharge so that I can improve not only myself but also my relationships with others.

###### Fortifying Mental Well-Being

- I have also noticed that I am more focused and organized in both my personal and work life.
- It's very recharging to recenter with TM.
- I feel more engaged in what I am doing.

###### Balancing Emotional Well-Being

- I also feel like TM has helped me to remain more calm, be more focused, and think clearer in stressful, emergency situations at work. I am able to respond very quickly and focus on exactly what is needed without feeling stressed or anxious.
- Being able to manage (emotions) internally/more effectively since doing TM.
- Definitely feel a sense of calm more often in everyday life.
- I’m more aware of how daily stress affects me physically and mentally, and how TM is helping relieve that stress … I’ve been able to see a reduction in how I hold on to stress in my mind and body.
- TM has helped me with the anxiety I always associated with socializing. Since practicing (TM) the internal chatter has softened and quieted.

##### Doing is living truth through cocreating a healing environment with others

###### Creating a Space for Calm and Healing

- I have been able to help my friends, family, and patients when they have anxiety or depression. I create a calming space for them to heal.
- The ability to be at peace with who I am and to have that calm project from me.
- I encourage others to take time for themselves … to breathe, meditate (to let thoughts come and go), put down their electronic devices (i.e., computers, mobile devices, etc.), and take a walk outside with nature. I try to share little things to help ease their sense of calmness and what it means to let things go and just be in the moment without interacting with anything.

###### Listening and Genuinely Connecting with Compassion

- Authentic presence is being engaged with either yourself, your surroundings, or others in multiple facets including physical presence, full attention of the mind, and creation of space.
- Being engaged with patients, which includes active listening, good eye contact, and expressing empathy in your words and in your actions.
- Yes, I just didn’t know what I have been experiencing is named authentic presence. That's it, not just hearing someone, actually stopping, and looking them in the eye and experiencing that moment in life together—good or bad-together. In this short life at this short meaningful moment together. Just as intended.
- I think I greet and welcome people more than before. I acknowledge their presence when passing by.
- TM has helped me slow down and pay more thoughtful attention to my patients, what they are saying and feeling. Listening has become more important and ensuring I am understanding what the patients feel they are going through.
- I share the mindfulness presence with other nurses that I mentor and teach.

###### Choosing Full Presence With Others in the Moment

- I try to be more “present” with my kids every day and feel like TM has helped me with this. Instead of feeling like I have to multitask the entire day, I purposefully have time for them when I don't have my phone or computer around.
- I make a point to be authentically present when engaging with others. I create the space and I show up with full mind and body.
- I engaged with a patient the other day using authentic presence. I came into his room to do his care. I could tell that he had been crying. I sat with him and let him talk to me about what he was feeling without interruption and without thinking of any tasks that I needed to complete. I focused on hearing him and being fully present for him.

###### Enhancing Emotional Intelligence to Transform Connections

- TM has helped me become more present with my children and more aware of how my interactions play a HUGE role in their responses to them.
- Instead of “going through the motions” I have been more honest with myself about how I really feel about things, and this has changed my interactions with people around me.
- Being able to be compassionate and caring for my patients without internalizing
- We give pieces of ourselves every day. Authentic presence however takes a different meaning now post TM practice. I have always been “stretched” thin and many times, I had found myself multitasking often. Most of the time “multitasking” meant no quality. In other words, I wouldn't be authentically present. TM has sparked a new way of seeing things. It is hard to explain, but the best way I can describe TM is “reflection followed by reorganizing.” Those thoughts during TM have an unexplained reason to be there but letting it happen and take its course has provided a “cleaner slate” so to speak and now I am able to prioritize and take presence to a deeper level with much more positive influence.
- TM has helped me become more authentically present with my children especially. I am more in tune with them than I ever have been and less distracted. This has given me more patience and appreciation for the everyday events and memories I'm making with them. I think the practice (taught to me) during meditation, has really taught me how to be authentically present with someone. I feel lucky to have learned TM and feel it should be a life skill everyone should learn to help with mental health and well-being. TM has helped me and my family, thank you!

##### Becoming is connection as universal truth through transcending self

###### More Spiritually Connected

- It (TM) has also created a deeper connection with my soul and the universe.
- Through TM I feel that I am able to connect more on an emotional and spiritual level with others both personally and professionally.
- The word flow comes to mind. There's understanding present.

###### Enhancing Gratitude

- … I do feel that my relationships in work and personal life are improving. I feel like I'm able to appreciate and enjoy people more.
- Practicing TM has made me more aware of my need to put the wasted energy of anxiety into enjoying the love and gratitude of life and family.
- I feel like I am open more to understanding that everyone has something going on that we can't see/be aware of—and feel more compassion and tolerance in general.
- Overall, TM has been a wonderful experience and a useful tool that I can carry forward in my life.

Qualitative findings were synthesized from these three concepts related to participants’ descriptions of their experiences when practicing TM: present moment awareness, domains of well-being, and authentic presence. The findings will be discussed in the context of Watson's (2018) theory and the published literature.

## Discussion

For participants in this study, the essence or meaning of the experience of practicing TM that surfaced is: *Authentic presence is veritas (truth) in knowing, being, doing, and becoming*. When viewing our qualitative findings through Watson's (2018) unitary caring science theory, we perceived nurses’ awareness at the moment (or knowing), as influencing their insight and value of well-being (doing), leading to an experience of authentic presence with self and others (being), in transforming their personal and professional lives (becoming). Participants shared descriptions of the positive impact that learning and practicing TM had on their authentic presence with others. The findings are congruent with our conceptual model—for example, present-moment awareness was enhanced, which impacted self-care and well-being, leading to ways that nurses engaged with others using authentic presence.

It is interesting, we believe, that through introducing TM to our participants and providing them with the opportunity to qualitatively share descriptions of their perceptions, our research findings and analysis were related to Watson's (2018) unitary caring science theory, (especially during the COVID-19 pandemic). During this time, Sitzman and Craven (2021) described the immediacy of the needs of nurses struggling with their ability to find connections physically (due to protective clothing) and emotionally (due to the stressors placed upon them in carrying on), amidst the fear and challenges of the pandemic. It was the visibility of the challenges that our clinicians faced which propelled and made possible funding opportunities to improve the health and well-being of our nurses, and in turn, to care for patients at an unprecedented time in our history.

### Augmenting the Literature

Our findings uncovered many similarities to Watson's (2008) 10 caritas processes (CPs). In this article, we will highlight CPs 1, 6, 8, and 10. It is noteworthy that our conceptual model and our findings could be viewed from the lens of Watson's (2018) unitary caring science theory, as we saw nurses’ ability to find stillness within as a critical element impacting nurses’ health and the way they are in the world with self and others.

The themes of knowing and being are very relevant to CP1: “Sustaining humanistic-altruistic values by practice of loving-kindness, compassion and equanimity with self/other” (Watson, 2018, p. 54). Our participants emphasized the subthemes of the necessity and value of present-moment awareness and being present with self/others without distraction (authentic presence). “Authentic presence in any given moment between persons captures the human-to-human spirit-to-spirit connection, which is felt experientially but may not be detected by an outside objective observer” (Watson, 2018, p. 90). What is remarkable is that our participants described their perspectives of authentic presence—those connections that they noticed after learning to meditate with TM. This is congruent with Perkins and Aquino-Russell's (2017) findings where “participants found themselves authentically present and balanced with enhanced job performance” (p. 163).

Participants described how their experience was enhanced through present moment awareness in the theme Being and subthemes: caring for the integrity of the self; prioritizing self-care for well-being; strengthening physical well-being; fortifying mental well-being; and balancing emotional well-being. This led to a greater focus on self-care as a personal gesture, perhaps a function of self-compassion. Watson's theory is consistent with other TM study findings which support the importance of nurses’ self-care (Aquino-Russell et al., 2023; Bonamer & Aquino-Russell, 2019; Perkins & Aquino-Russell, 2017; Schenosky, 2023). Congruent with our findings, Schenosky (2023) found that TM practice as a self-care intervention among nursing students was effective in enhancing physiological and psychological health during the COVID-19 pandemic with qualitative reports of improved motivation, confidence, compassion, and overall calmness. Aquino-Russell et al. (2023) reported similar results among nursing students and nursing instructors with a qualitative study uncovering TM's positive impact on participants’ lives for managing stress, enhancing productivity, and improving relationships. Bonamer and Aquino-Russell (2019) reported that the practice of TM among clinical nurses was shown to improve resilience and reduce compassion fatigue. Watson (2008) wrote, “Caring begins with being present, open to compassion, mercy, gentleness, loving-kindness, and equanimity toward and with self before one can offer compassionate care to others” (p. xviii). Nurses’ descriptive responses emphasized caring for self as an important step toward wholeness thus enhancing their being with others and allowing them to engage more intentionally.

Our theme of doing emphasizes the authentic act of caring for others as nurses, who are aware of living the subthemes of creating a space for calm and healing; listening and genuinely connecting with compassion; choosing full presence with others in the moment; and enhancing emotional intelligence to transform connections. These actions relate to CP6, which is “Creatively problem-solving- ‘solution-seeking’ through caring processes” (Watson, 2018, p. 56)*; “*full use of self and artistry of caring-healing practices via use of all ways of knowing/being/doing/becoming” (Watson Caring Science Institute (WCSI), 2023, np). Our participants’ reports of enhanced emotional intelligence from TM practice are consistent with the research reported by Valosek et al. (2019).

When authentically engaged in caring for others in providing a healing environment, nurses become the healing environment, consistent with CP8, which is, “Creating a healing environment at all levels; subtle environment for energetic authentic caring presence” (Watson, 2018, p. 56). Watson emphasizes the importance of the nurse as the environment (Quinn, 1992). TM helped participant nurses to live caring for themselves and others, with an increased emphasis on self-care. Further, nurses shared an enhanced sense of emotional intelligence which allowed them to distinguish between self and others. With TM, nurses described serving as the healing environment in the moment, creatively employing all aspects of self as caring instruments without absorbing the struggles of others. TM, as an expression of self-care, serves to reinforce the requirement of holistic nursing in being a healing instrument (ANA & AHNA, 2019). “Caring as an ethical ideal” is the essence of professional nursing practice and is what nurses offer society (ANA, 2021; Watson, 2018, p. 7). The cocreation of caring connections in the healing environment led to participant nurses’ experience of becoming.

Nurse participants reported TM as encouraging a sense of becoming, including subthemes of enhanced spiritual connectedness and gratitude. This is congruent with CP10 “Opening to spiritual, mystery, unknowns-Allowing for miracles” (Watson, 2018, p. 56). Nurses experienced a profound understanding of the sacredness and connectedness of us all. Watson (2008) described this openness to transcendence as a “higher plane of seeing the world” (p. 195). The experience of self is universal to all persons and yet remains a mysterious phenomenon (Watson, 2008). There is magic in the exploration of self and connection to others and the universe. Perkins and Aquino-Russell (2017) reported participants’ enhanced health, healing, and well-being, as well as “Feelings of peacefulness, bliss, and integrity … which enhanced the experience of sacred space amidst everyday stressors, while compassion, gratitude, grace, appreciation, and care were inherent within” (p. 163). Our participants also described presence, authenticity, integrity, and caring in relationships which facilitated appreciation of “the good, the true, and the beautiful” in others (McIntosh, 2015, p. 53). In alignment with this sense of enlightened becoming, Watson (2018) proposed:

The future of care will be soul care … That is what is missing in our dominant medical systems. … Nursing has such a critical role to play in ‘ensouling’ the healthcare system with new models of caring-healing whereby the artistry of our divine being is built into consciousness. (p. 102)

This continuum of knowing, being, doing, and becoming is embedded within authentic presence which resulted from nurses’ practice of TM.

Our study's findings (participants’ descriptions) were consistent with Watson's (2018) concept of authentic presence of caring with self and others. Themes from published literature describing authentic presence with others, included “(a) authentic presence requires a commitment to the caring moment; (b) authentic presence reflects an intentional connection with others; (c) authentic presence promotes healing with compassion” (Pullyblank, 2023, p. 158). To date, no referrals to authentic presence with self were found in the literature. Our participants described self-care, self-acceptance, and self-compassion as manifestations of authentic presence with self.

Our study's qualitative results have uncovered similarities to other researchers’ quantitative findings. Our participants perceived enhanced present-moment awareness and diminished anxiety and stress. According to Barnes et al. (2001), MacLean et al. (1997), and Walton et al. (2004), the TM technique has positive effects in reducing psychological and physiological responses to stressors, as well as reducing elevated cortisol levels. Travis et al. (2009) found that TM promoted coherent brain functioning, associated with lower stress levels. Furthermore, consistent with other published research on clinicians and students, improvements were seen with reductions in psychological symptoms (Azizoddin et al., 2021; Bonamer et al., 2024; Nestor et al., 2023; Rosaen & Benn, 2006; Travis, 2013; Vela-Valenzuela et al., 2021; Wendt et al., 2015), and increased resilience (Bonamer & Aquino-Russell, 2019; Wendt et al., 2015).

Despite this study occurring during the COVID-19 pandemic, nurses’ responses did not particularly focus specifically on the impact of TM in relation to their experiences with COVID-19, but rather the influence of TM more broadly as a strategy for personal and professional well-being. The latter finding is consistent with recently published TM research among clinicians demonstrating improvements in mental well-being (Calarco & Stratton, 2023). Further, even though our participants were novice meditators, they described profound experiences and results, which are consistent with seasoned meditators (Perkins & Aquino-Russell, 2017). Our clinical nurse participants expressed gratitude for the opportunity to learn TM, which is congruent with Aquino-Russell et al.'s (2023) and Perkins and Aquino-Russell's (2017) participants’ perspectives.

This study illuminates the development of a unique multidimensional conceptual model of nurses’ well-being. While scant published evidence describes the benefits of TM among nurses, this study adds to the literature by proposing a model which defines the complexity of nurse well-being and proposes a strategy (i.e., TM) which impacts the multiple dimensions of nurse well-being. Further, this study offers insight into clinical nurses’ experiences with well-being during the COVID pandemic.

### Strengths and Limitations

It is noteworthy that the three hospitals included in this study were all Magnet-recognized, which may be demonstrated in the presence of onsite nurse researchers, a nursing culture of professional development, and a willingness on behalf of the organizations to support self-care programs for nurses. Although Magnet recognition does not directly include self-care promotion, the Magnet culture of nursing excellence may have contributed to the commitment among nurses to participate (American Nurses Credential Center, 2024). This is consistent with the large volume of participant survey responses received from all participants who completed TM instruction (100%, *n* = 46).

This research builds on nursing knowledge as the findings were viewed through the lens of a nursing theory (Watson, 2018). A limitation of our study is the inability to explore the process unfolding as nurses developed their TM technique, as we only have participants’ descriptions which were written following three months of practice. Nurses who were not randomized to the immediate instruction group were not provided with any information about how to receive TM instruction until the completion of their 3-month survey. However, we did not collect information specifically on the potential for comingling among colleagues.

## Conclusions

TM facilitated integration of nurses’ knowing, being, doing, and becoming leading to healing of self, and enhanced connections with and caring for others (authentic presence). Nurses shared experiences of personal growth which led to improvements in the ways that they engaged with others, including family members and patients. Participants’ responses were consistent with our conceptual model in that present-moment awareness prompted an increased emphasis on self-care and well-being, which meaningfully influenced the way nurses were authentically present with others. For Watson (2018),

The praxis of unitary caring science makes explicit the underlying values, ethics, as part of the entire single unitary field of human-earth-universe. It moves Caritas Processes of Praxis to embrace *Veritas* [italics added], the Latin word for *truth* [italics added], beauty, love, goodness, restoring the moral component within the full meaning of Praxis. Caritas and Veritas combine in unitary caring science, returning nursing to its underlying purity and purpose of basic goodness. These underlying values are needed today to offer a New Story of science and actions that can help sustain our humanity and planet earth. (p. 7)

The TM for Nurses Program utilizes evidence-based standardized instruction. This allows for greater ease in scaling TM instruction for larger audiences. The uniformity of TM instruction reduces concerns about the reliability and validity of the program, thereby improving the generalizability of findings.

### Implications for the Science of Holistic Nursing

This study has advanced Watson's (2018) holistic unitary caring science theory as it reinforces the presence and value of *Veritas,* the Latin word for *truth,* within the CPs of holistic nursing praxis. The nurses reflected on the journey of their TM practice as a path toward the evolution of the CPs with Veritas in knowing, being, doing, and becoming, enlivening self-care (a core value of holistic nursing practice).

### Implications for Holistic Nursing Practice

Our qualitative analysis of clinical nurses practicing TM during the COVID-19 pandemic provided illumination of the value of TM for supporting nurses’ multifaceted well-being with the goal of retaining nurses in practice. TM, as a recommended meditation strategy, is consistent with the core value of self-care for holistic nursing practice (ANA & AHNA, 2019). TM provides nurses with a mechanism for self-awareness, self-care, and self-healing in recognition of the complexities of their multidimensional health. When nurses care for self, they are more likely and able to care for others, thus helping them to enjoy their nursing careers. Future research may examine the impact of holistic nurses who practice TM on patient outcomes.

The American Heart Association recommends meditation practices, such as TM, as a strategy to boost health well-being and decrease blood pressure (AHA, 2023; Brook et al., 2013). The American Nurses Association\California (2022) has identified TM as one of the top five “Best Fit” mental health services and solutions (np). Congruent with our study involving nurses, other organizations across the United States have introduced TM to physicians and other healthcare professionals with positive results related to burnout, resilience, and well-being, especially during the COVID-19 pandemic (David Lynch Foundation, Center for Resilience, 2023).

Also, TM instruction to clinical students has been introduced as a proactive strategy to assist them in their learning and future healthcare careers. For example, a medical school in the United States has integrated TM into their curriculum, so that physicians could work towards healing themselves (Loiselle et al., 2023; Nader et al., 2023), while a university in Canada introduced TM to nursing students and faculty members (Aquino-Russell et al., 2023).

We highly recommend that academic and healthcare organizations support clinical nurses by teaching TM and encouraging/supporting regular practice. Clinical nurses in our study shared their experiences with the practice of TM in creating present-moment focus, leading to enhanced self-care and the development of authentic presence with others.
